# The evolution of masturbation is associated with postcopulatory selection and pathogen avoidance in primates

**DOI:** 10.1098/rspb.2023.0061

**Published:** 2023-06-14

**Authors:** Matilda Brindle, Henry Ferguson-Gow, Joseph Williamson, Ruth Thomsen, Volker Sommer

**Affiliations:** ^1^ Department of Anthropology, University College London, 14 Taviton Street, London WC1H 0BW, UK; ^2^ The Institute of Zoology, Zoological Society of London, Regent's Park, London NW8 7LS, UK; ^3^ Centre for Biodiversity and Environment Research, Department of Genetics, Evolution and Environment, University College London, Gower Street, London WC1E 6BT, UK; ^4^ School of Biological and Chemical Sciences, Queen Mary University of London, London E1 4NS, UK

**Keywords:** masturbation, postcopulatory sexual selection, pathogen avoidance, Bayesian phylogenetics

## Abstract

Masturbation occurs throughout the animal kingdom. At first glance, however, the fitness benefits of this self-directed behaviour are unclear. Regardless, several drivers have been proposed. Non-functional hypotheses posit that masturbation is either a pathology, or a byproduct of high underlying sexual arousal, whereas functional hypotheses argue an adaptive benefit. The Postcopulatory Selection Hypothesis states that masturbation aids the chances of fertilization, while the Pathogen Avoidance Hypothesis states that masturbation helps reduce host infection by flushing pathogens from the genital tract. Here, we present comprehensive new data documenting masturbation across the primate order and use these, in conjunction with phylogenetic comparative methods, to reconstruct the evolutionary pathways and correlates of masturbation. We find that masturbation is an ancient trait within the primate order, becoming a more common aspect of the haplorrhine behavioural repertoire after the split from tarsiers. Our analyses provide support for both the Postcopulatory Selection and Pathogen Avoidance Hypotheses in male primates, suggesting that masturbation may be an adaptive trait, functioning at a macroevolutionary scale.

## 1. Background

Autosexual behaviour, or masturbation, is common across the animal kingdom, but appears to be particularly prevalent in the primates [1,2]. There is little systematic comparative research into this behaviour, and its evolutionary history is unclear. The proximate driver of masturbation is not difficult to establish, since genital stimulation is reinforced through the same hedonic feedback [3,4] regardless of whether it is auto- or allosexual. However, at a superficial level, masturbation poses a problem for evolutionary theory. It does not directly increase survival prospects and, by definition, occurs to the exclusion of reproductive partners, while incurring costs in terms of time, attention and energy. Consequently, masturbation has historically been considered, at worst, a pathological behaviour carried out by aberrant, typically captive, individuals and, at best, a sexual outlet necessitated by high libido [5]. However, the Pathology Hypothesis fails to account for autosexuality in wild-living primates. The Outlet Hypothesis may not account for masturbation immediately pre- or postcopulation, or in the presence of willing partners, particularly in males of species with an extended refractory period (i.e. the period of time after ejaculation, where sexual arousal and activity is reduced [3]). In any case, the idea that masturbation may serve as a sexual outlet is not mutually exclusive with adaptive explanations, since arousal can be understood as the proximate mechanism of the indirect ultimate causes (i.e. functions).

The Postcopulatory Selection Hypothesis [1,2,6] predicts that masturbation aids the chances of fertilization and is composed of two constituent hypotheses: the Sexual Arousal Hypothesis and the Sperm Quality Hypothesis. The Sexual Arousal Hypothesis posits that non-ejaculatory male masturbation may speed up subsequent ejaculation, benefiting low-ranking males in danger of being outcompeted by rivals (e.g. in marine iguanas, *Amblyrhynchus cristatus* [7]; and Japanese macaques, *Macaca fuscata* [8]), or increase ejaculate quality (e.g. in humans, *Homo sapiens*, [9]). Similarly, the Sperm Quality Hypothesis states that ejaculatory precopulatory male masturbation may expel inferior sperm, improving subsequent ejaculate quality (e.g. in humans [10]; Japanese macaques [8]; and Rhesus macaques, *Macaca mulatta* [11]). The Sexual Arousal Hypothesis could also help explain female masturbation, since sexual arousal and orgasm may facilitate cryptic female choice. Female arousal increases vaginal pH, creating a more hospitable environment for sperm [12]. The vaginal transudate associated with arousal filters out inferior sperm, while facilitating the transfer of high-quality sperm towards the uterus [13]. Similarly, the contractions associated with female orgasm may enhance the passage of sperm through the uterine cavity, and associated secretions of prolactin capacitate sperm [13,14]. While little is known about the storage and survival durations of sperm within the female reproductive tract in non-human primates, in humans, sperm can be viable for greater than 5 days [15,16]. It seems likely, therefore, that female primates could use pre- or postcopulatory masturbation as a strategy to increase their chances of being fertilized by a given male. Alternatively, masturbation could also serve as a form of precopulatory display or courtship behaviour in both sexes [2,17], similar to precopulatory ‘penile displays' in chimpanzees [18].

The Pathogen Avoidance Hypothesis predicts that masturbation is a form of postcopulatory genital grooming, helping to prevent sexually transmitted infections (STIs). Behavioural strategies are often the first line of defence against infection, followed by physiological and immune responses [19]. Postcopulatory grooming strategies, such as oral self-cleansing or urination, have been adopted by various mammals (fruit bats, *Cynopterus sphinx* [20]; certain callitrichid and strepsirrhine primates [19]; humans [21]; and murine rodents [22]). An alternative strategy is used by male Cape ground squirrels (*Xerus inauris*), who masturbate after mating, cleansing the reproductive tract with ejaculate [17].

We employ Bayesian phylogenetic Markov chain Monte Carlo (MCMC) models and hierarchical modelling to reconstruct the evolutionary history of masturbation and test the basic predictions of two functional hypotheses (the Postcopulatory Selection Hypothesis and Pathogen Avoidance Hypothesis) within a comparative framework for the first time, using a large-scale dataset charting masturbation across the primate order. The Postcopulatory Selection Hypothesis predicts that masturbation should be associated with high postcopulatory selection pressure, as measured through the proxies of multi-male mating systems and relative testes mass. The Pathogen Avoidance Hypothesis predicts that taxa are more likely to masturbate if exposed to a higher pathogen load, as measured via the proxies of geographic region, mean annual precipitation and environmental harshness, as well as the more direct measure of STI load.

## 2. Results

### (a) Data assembly

We assembled the largest dataset of masturbation occurrence to date, compiled from nearly 400 sources, including 246 publications and 150 questionnaire responses and personal communications from primatologists and zoo-keepers [1,2]. We combined this dataset with a posterior distribution of 10 000 molecular phylogenies spanning the primate order, from the 10k Trees Project [23]. Our data covered 105/272 species (38.6%), 54/67 genera (80.6%) and 18/19 (sub)families (94.7%) within our phylogeny. The proportion of species from each major radiation for which we have data varies, and likely reflects a research bias towards larger-bodied primates. Thus, data were available for 19.7% of lemurs, 33.3% of lorises and galagos, 20.0% of tarsiers, 41.8% of platyrrhine monkeys, 40.4% of catarrhine monkeys and 68.2% of apes.

The data cover both sexes, with data for females covering 67.2% of genera (*n*_species_ = 49), and males covering 76.1% of genera (*n*_species_ = 83). This allowed us to run all analyses separately for females and males, to account for potential sex differences.

Both wild and captive primates are represented in our dataset, though reports of masturbation are more common for captive primates (*n* = 298) than wild individuals (*n* = 105). However, given that many species are more commonly studied in captivity, the absolute number of reports is not necessarily informative. A more appropriate measure is to compare how many studies on captive versus wild primates report masturbation, or the lack thereof. In our dataset, masturbation is reported to be present in 74.5% of studies on captive females, and 87.4% of studies on captive males, versus 35.4% of studies on wild females, and 73.3% of studies on wild males.

### (b) Phylogenetic signal and ancestral state reconstructions

Both female and male masturbation showed strong phylogenetic signal indicating that species with more recent shared ancestry have more similar trait values (electronic supplementary material, table S1).

**Figure 1.**
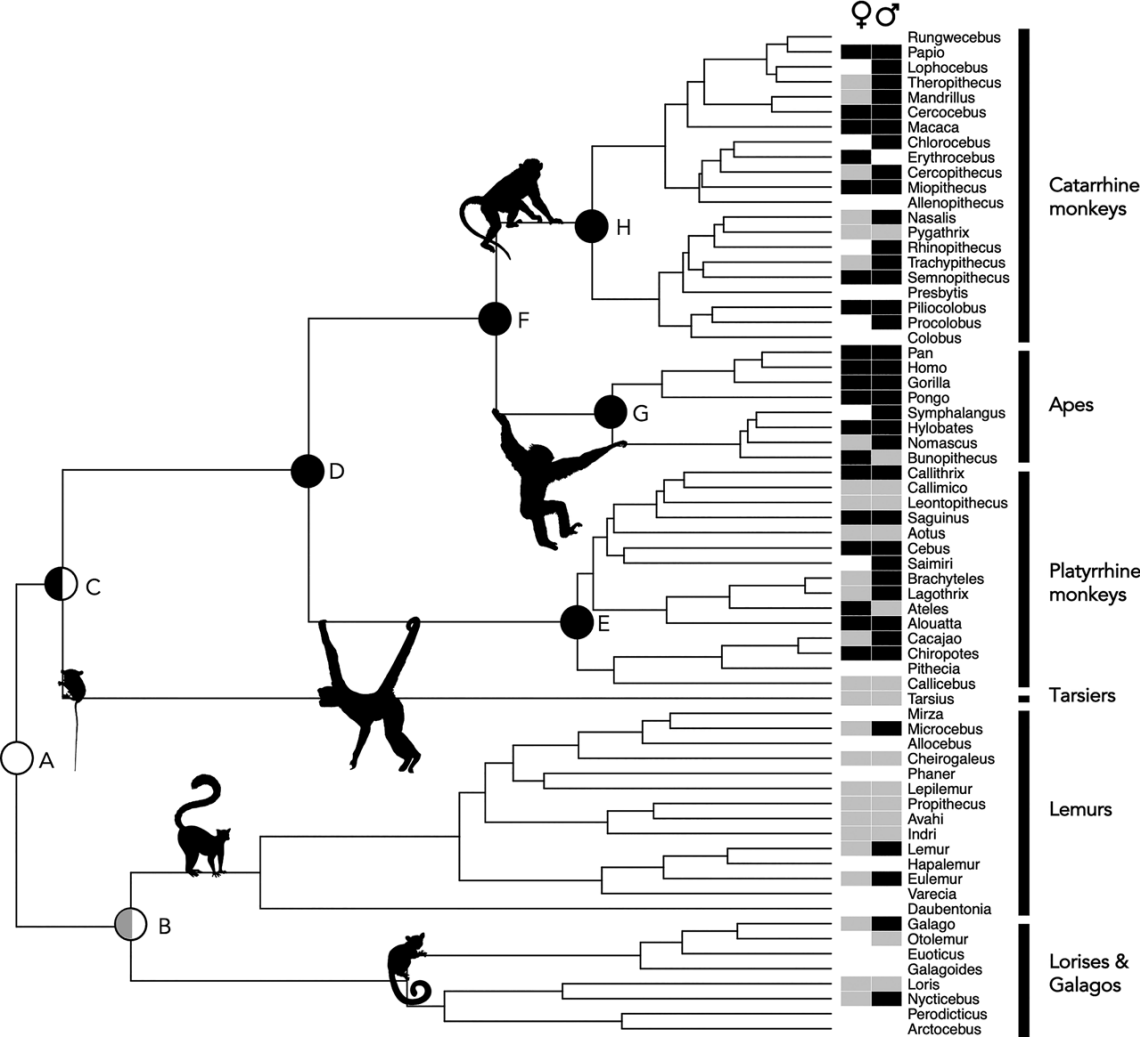
Phylogeny of 67 genera of the primate order, illustrating reconstructed ancestral nodes and traits of extant genera in female and male primates (electronic supplementary material, table S2). Reported presence (black squares) or absence (grey squares) of masturbation in extant females (♀) and males (♂) in at least one species of that genus is indicated at the tips of the tree. Empty tips indicate a lack of data. At the nodes of the tree, black shading indicates masturbation presence, grey shading indicates masturbation absence (both mean probability greater than 0.60), and white indicates equivocal reconstructions (mean probability less than 0.60). Ancestral state reconstructions showed broadly the same pattern in female and male primates, with the exceptions of node C, which indicated masturbation was present in females (left half of circle) but was equivocal for males (right half of circle), and node B, which indicated that masturbation was absent in females (left half of circle) but was equivocal for males (right half of circle). Maximum clade credibility tree created from a sample of 10 000 molecular phylogenies from the 10kTrees project [1].

Ancestral state reconstructions of female masturbation (electronic supplementary material, table S2; figure 1; Bayesian rjMCMC ancestral state reconstructions; *n*_species_ = 49) indicate that masturbation was present in ancestral (non-tarsier) haplorrhines (node D; mean probability = 0.76), catarrhines (node F; mean probability = 0.91), apes (node G; mean probability = 0.95) and catarrhine monkeys (node H; mean probability = 0.87). Other nodes were reconstructed with less confidence, with the model indicating that masturbation was probably present in the ancestral haplorrhine (including tarsiers; node C; mean probability = 0.65) and platyrrhine monkey (node E; mean probability = 0.63), but absent in the ancestral strepsirrhine (node B; mean probability = 0.67). The estimation of female masturbation occurrence at the root was equivocal (node A; mean probability of masturbation presence = 0.55).

Ancestral state reconstructions of male masturbation (electronic supplementary material, table S2; figure 1; Bayesian rjMCMC ancestral state reconstructions; *n*_species_ = 83) followed the same broad pattern. Masturbation was present in ancestral (non-tarsier) haplorrhines (node D; mean probability = 0.74), catarrhines (node F; mean probability = 0.92), apes (node G; mean probability = 0.95) and catarrhine monkeys (node H; mean probability = 0.97). The ancestral platyrrhine monkey node was reconstructed with less confidence, but masturbation was likely present (node E; mean probability = 0.67). Compared to females, ancestral male haplorrhines (including tarsiers; node C; mean probability of masturbation presence = 0.58), and strepsirrhines (node B; mean probability of masturbation presence = 0.49), were reconstructed with lower confidence. In keeping with females, the estimation of male masturbation occurrence at the root was equivocal (node A; mean probability of masturbation presence = 0.56).

### (c) Postcopulatory selection hypothesis

Different mating systems vary in the level of postcopulatory selection and associated behavioural and morphological phenotypes they generate [24]. For example, multi-male mating systems produce high levels of postcopulatory male–male competition, and are associated with large testes to body mass ratio [5,25] and elongated bacula [24]. We therefore employ mating system and testes mass (controlled for body size) as proxies for postcopulatory selection, using the compilation of data in Brindle & Opie [24].

**Figure 2.**
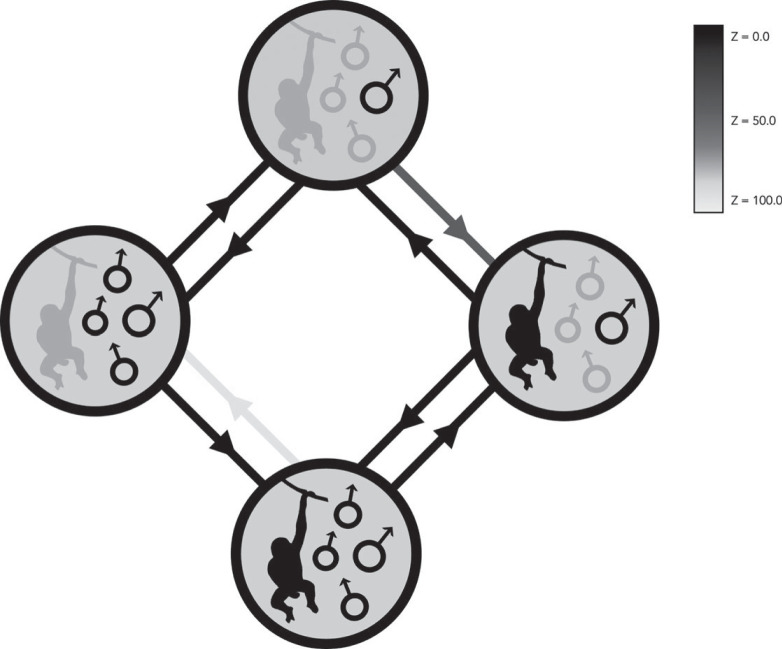
Coevolution between masturbation occurrence (present versus absent; left-hand side of circles in schematic) and mating system (single- versus multi-male, right-hand side of circles in schematic). Traits present in a given state are shown in black, and those absent are shown in grey. ‘Z' denotes the percentage of models where a transition did not occur, which indicates how likely a transition is to have taken place. Black arrows correspond to transitions with a very low posterior probability of being zero (i.e. very common transitions), dark grey arrows to a low posterior probability of being zero (i.e. common transitions), and light grey arrows to a high posterior probability of being zero (i.e. rare transitions).

We found support for coevolution between masturbation presence and mating system in male, but not female, primates, providing support for the Postcopulatory Selection Hypothesis in males (electronic supplementary material, table S3; figure 2; rjMCMC models of correlated versus independent evolution; *n*_species_ = 299; male masturbation and mating system, log BF = 2.44; female masturbation and mating system, log BF = −2.40). Examination of evolutionary transitions revealed that, in male primates, shifts from masturbation absence to presence occurred in both single- and multi-male mating systems (*Z* = 18.8% and 0.2%, respectively; where *Z* is the percentage of models where a transition did not take place, indicating the likelihood of a given transition), though more frequently in multi-male mating systems. However, masturbation was also lost frequently in single-male mating systems (*Z* = 0.0%), but almost never in multi-male mating systems (*Z* = 98.7%). This indicates that—while masturbation is a very labile trait in single-male mating systems—in multi-male mating systems, once male masturbation has evolved, it persists.

**Figure 3.**
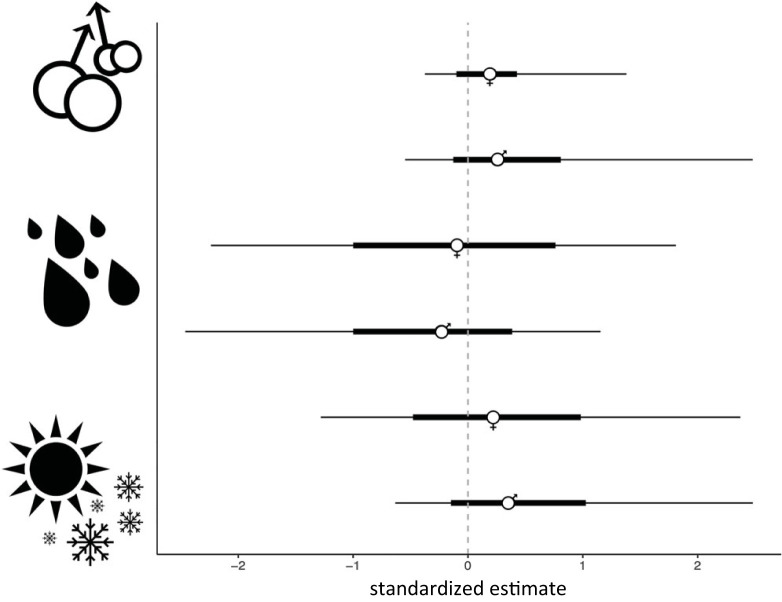
Standardized estimates (estimate/error) for masturbation presence in female and male primates with adult male testes mass (top), mean annual precipitation (middle) and environmental harshness (bottom). Points indicate posterior means, thick horizontal lines indicate 80% Bayesian credible intervals (BCIs), and thin horizontal lines indicate 95% BCIs.

We did not find evidence for a relationship between masturbation and adult male testes mass in either female or male primates (electronic supplementary material, table S4; figure 3).

### (d) Pathogen avoidance hypothesis

Many parasites require hot, humid conditions or water, to complete their life cycles. This may explain why global parasite diversity tends to increase towards the equator, and is closely linked to climatic variables [26,27]. For this reason, we employ (i) primate geographical region (temperate versus tropical), (ii) mean annual precipitation, and (iii) environmental harshness (the degree of climatic predictability) as a proxy for pathogen load. We also invoke the more direct measure of sexually transmitted pathogen occurrence in wild populations, gleaned from the Global Primate Parasite Database (GPPD) [28], which was categorized as either present or absent for a given species.

**Figure 4.**
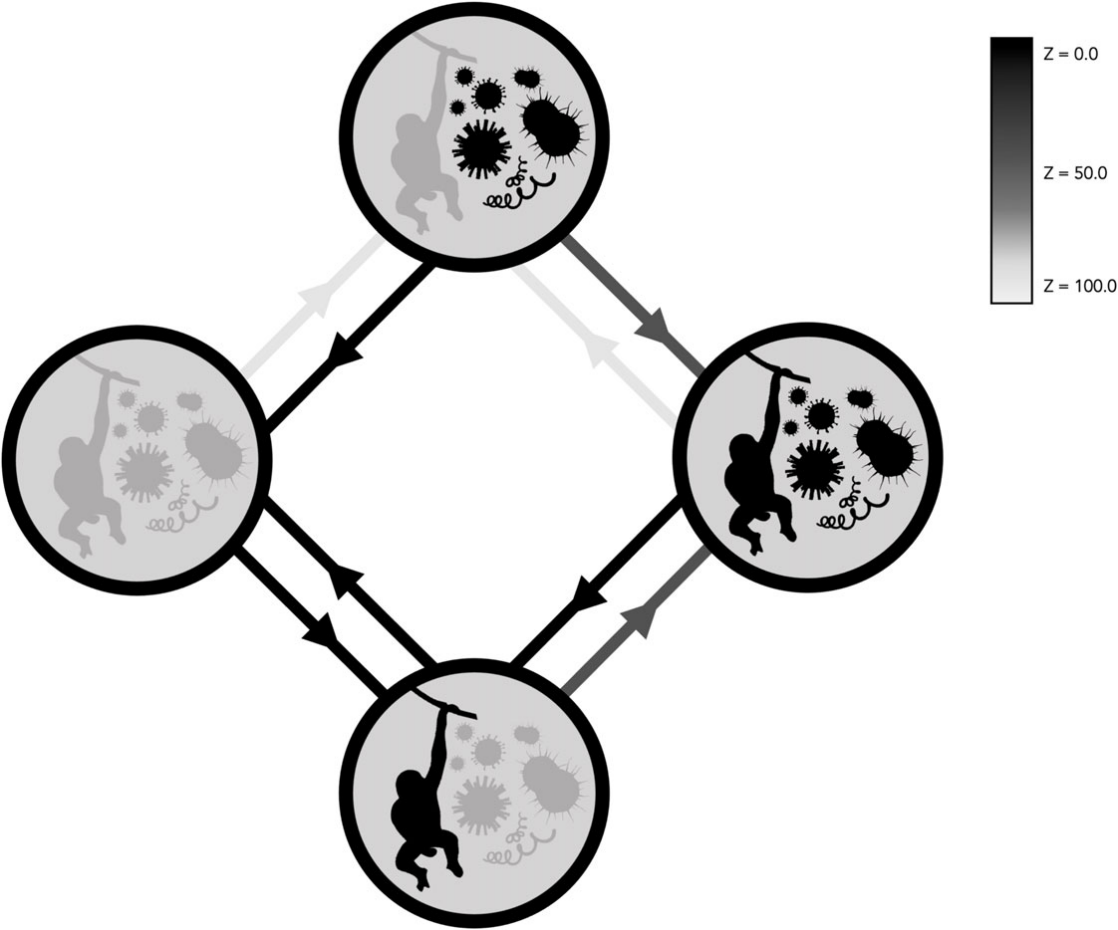
Coevolution between masturbation occurrence (present versus absent; left-hand side of circles in schematic) and pathogens (present versus absent; right-hand side of circles in schematic). Figure 2 for further details.

We found very strong evidence for coevolution between masturbation and pathogen occurrence in males, but not females, providing support for the Pathogen Avoidance Hypothesis in males (electronic supplementary material, table S3; figure 4; rjMCMC models of correlated versus independent evolution; *n*_species_ = 299; male masturbation and pathogen occurrence, log BF = 11.25; female masturbation and pathogen occurrence log BF = – 1.22). Evolutionary path analyses indicate that, in male primates, shifts from masturbation absence to presence occurred regardless of whether pathogens were absent or present (*Z* = 0.6% and 26.7%, respectively). However, masturbation was lost at a very high rate when pathogens were absent (*Z* = 0.0%) but almost never when they were present (*Z* = 99.8%). This indicates that masturbation is a labile trait when pathogens are absent, but when pathogens are present masturbation is retained.

By contrast, our analyses with respect to environmental predictors of pathogen risk failed to find any patterns. We found no evidence in support of coevolution between masturbation and geographical region in female or male primates (electronic supplementary material, table S3). Similarly, there was no support for a relationship between masturbation and mean annual precipitation or environmental harshness, in either females or males (electronic supplementary material, table S4; figure 3).

To confirm that our results were not an artefact of potential coevolution between pathogen presence and mating system, we also checked these variables for coevolution, but did not find support for this model (electronic supplementary material, table S3).

## 3. Discussion

While some consider masturbation to be pathological (e.g. [29]) or a byproduct of neuroendocrinological specialization for high sexual arousal [5], others stress its adaptive role in postcopulatory selection [1,2,6,30] or STI reduction [17]. The data presented here highlight the inadequacy of the Pathology Hypothesis to explain autosexuality in primates since, in our dataset, masturbation is reported in over a third of studies on wild females, and over two-thirds of reports on wild males.

Our analyses demonstrate that masturbation has a strong phylogenetic signal and is an ancient trait within the primate order. We also find that both postcopulatory selection and pathogen presence have been associated with masturbation across the course of evolution in male, but not female, primates.

With regards to adaptive explanations, we found support for the Postcopulatory Selection Hypothesis in male primates, with masturbation covarying with mating system, but not testes mass. Male masturbation presence is unstable in single-male mating systems, but evolves and is maintained in multi-male mating systems. We also found strong support for the Pathogen Avoidance Hypothesis in males, with masturbation coevolving with pathogen presence. Similar to the results for male masturbation and mating system, when pathogens are absent, male masturbation presence is labile, but when they are present, masturbation evolves and is retained.

There are several explanations for why masturbation could coevolve with pathogen presence in males but not females. One of the key functions of seminal plasma is to provide antibacterial defence for sperm once it reaches the female reproductive tract [31], but these bactericidal properties also protect hosts from STIs [32]. In males, ejaculate is forcefully expelled through the urethra, whereas during female arousal, vaginal transudate passes gradually through the vaginal wall, coalescing to form a film which is eventually secreted [33]. Since the urethra is the primary site of infection for many STIs [21], the powerful expulsion of bactericidal ejaculate through the urethral opening is likely to be a more effective means of avoiding infection than the slow secretion of transudate from the vaginal canal, which does not necessarily cleanse the urethra. Furthermore, unlike in males, the Postcopulatory Selection Hypothesis and Pathogen Avoidance Hypothesis may be mutually exclusive in females. Under normal conditions, when a female is not aroused, vaginal pH is moderately acidic, in order to defend against infection [12]. However, according to the Postcopulatory Selection Hypothesis, one mechanism of cryptic female choice is that, during arousal, vaginal pH increases to become more neutral. While this creates a more hospitable environment for sperm, allowing females to differentially favour certain males, it is also less hostile towards pathogens, leaving females vulnerable to infection [12].

What is more surprising is that we did not find support for the Postcopulatory Selection Hypothesis in females. Neither female nor male masturbation showed an association with adult male testes mass, in contrast to previous findings [5], and female masturbation did not coevolve with mating system. The discrepancy between our results and previous research may be due to our introduction of a phylogenetic framework, in conjunction with a larger sample size, highlighting the importance of using rigorous phylogenetic methods for comparative research. Regardless, this does not explain why male masturbation has been associated with multi-male mating systems across the course of evolution, but female masturbation has not. It is important to note that there are far fewer reports for masturbation in female primates in our dataset. This is in part because female arousal and masturbation can be less conspicuous than that of males, but also reflects a broader paucity of information on female sexual behaviour and morphology in the biological sciences. While it is possible, therefore, that female masturbation is not associated with a high degree of postcopulatory selection pressure, it is important to gather more data to fully assess this hypothesis.

The results presented here are consistent with the idea that primates have evolved behavioural strategies, in this case masturbation, to reduce the risk of STIs. However, a previous study [34] found no evidence that species at greater risk of infection (i.e. primates that mate with multiple partners, as defined by relative testes mass and oestrus duration) were more likely to employ postcopulatory strategies to cleanse the reproductive tract: oral–genital grooming and urination [34]. Instead—while most strepsirrhines and callitrichids show stereotypic oral–genital grooming and urination after copulation—such behaviour is rarely seen in larger primates (catarrhines and non-callitrichid platyrrhine monkeys), perhaps because they are less able to reach their genitals orally. This may indicate that these traits are not environmentally driven, but reflect derived phenotypes. Interestingly, strepsirrhines and callitrichids also engage in scent-marking behaviours, including urination and anogenital rubbing [19], while masturbation is rare in these clades. It therefore seems possible that strepsirrhines and callitrichids are predisposed to urinate and orally groom their genitals, rather than masturbate, in order to mitigate STI risk. By contrast, in the absence of scent-marking and oral–genital grooming, larger primates may use masturbation to reduce the risk of STIs. If masturbation had already evolved in this group alongside increased postcopulatory selection pressure, it may have subsequently been exapted for pathogen avoidance (and maintained via the combined influence of postcopulatory selection and selection for pathogen avoidance). Indeed, ancestral state reconstructions indicate that masturbation was present in ancestral haplorrhines—after the split from tarsiers—and persisted in ancestral catarrhines and all subsequent nodes (apes and catarrhine monkeys). It is also likely that ancestral platyrrhine monkeys masturbated, although this node was less well resolved, particularly in females. However, the models suggested that masturbation was likely absent in ancestral female strepsirrhines, although they were equivocal regarding males.

Phylogenetic approaches can only capture a glimpse of the past, and the view can be incomplete or misleading when based only on present day information [35]. Our dataset contains less information for strepsirrhines compared with haplorrhines and, as such, the ancestral state of masturbation occurrence in the strepsirrhines was less well resolved than for clades with more data. Similarly, of the non-tarsier haplorrhines, there are fewest data for platyrrhine monkeys and, correspondingly, their ancestral state is reconstructed with a lower probability than other nodes. It may be that the lack of data in these taxa reflects the fact that absence data are less likely to be reported. Regardless, the uncertainty surrounding the strepsirrhine ancestral state has likely had a knock-on effect on estimates of the root of the phylogeny, which are also equivocal. The lack of certainty in these reconstructions is also likely to reflect the fact that the basal primate is the most phylogenetically distant from extant taxa, which has an adverse effect on ancestral state reconstructions. Gathering more data on strepsirrhines, therefore, would be an important step towards resolving deeper nodes within the phylogenies.

While there was strong support for the Pathogen Avoidance Hypothesis when examining pathogen load, no support was found when indirect measures of STI risk were used, namely geographical region, mean annual rainfall and environmental harshness. The most likely reason for this is that environmental predictors are poor indicators of STI risk. Although there is good evidence that such environmental variables are correlated with pathogen diversity (e.g. [26,27]), this may be driven by protozoan parasites [26], which are primarily transmitted via insect vectors. Since STIs are passed on via direct contact between individuals [36], the environmental measures employed as proxies here may not have adequately captured regional variation in STI risk. It is also possible that pathogen load is associated with other variables that may influence masturbation. For example, there is a positive relationship between host group size and the prevalence of directly transmitted parasites across taxa [37]. It would therefore be instructive for future research to explore the potentially complex relationships between masturbation, pathogen load and other factors associated with group-living in primates.

It is also worth noting that, although we found strong support for coevolution between male masturbation and pathogen pressure, categorizing pathogen load as ‘present' or ‘absent' in a given species is highly reductive. Similarly, masturbation rate and occurrence vary both within- and between-species, so that some individuals masturbate far more frequently, and some species far more ubiquitously, than others. In reality, both pathogen load and masturbation occurrence fall along a broad continuum. It would be useful to examine whether primates with a higher pathogen load are more likely to masturbate. With additional data, this question may be addressed in a more nuanced light.

Our findings provide a promising indication that masturbation may serve an adaptive function. We highlight important avenues of future enquiry regarding the evolution of primate masturbation that likely require empirical ‘ground truthing' via observational studies in a range of species, in addition to further comparative analyses. For example, to test between the Postcopulatory Selection Hypothesis and the Pathogen Avoidance Hypothesis in a given species, it is important to know whether masturbation occurs before (Postcopulatory Selection Hypothesis) or after (Pathogen Avoidance Hypothesis) copulation. Similarly, support for the Postcopulatory Selection Hypothesis in male primates does not allow us to distinguish the relative importance of two of the hypotheses it is underpinned by, namely, the Sexual Arousal Hypothesis and the Sperm Quality Hypothesis. To test between these, we need to establish whether masturbation occurs with ejaculation (Sperm Quality Hypothesis) or without (Sexual Arousal Hypothesis). Finally, although we did not find evidence in support of the Pathology Hypothesis, it would be informative to compare within-species masturbation rates in wild and captive individuals, to assess the relative effect of captivity on masturbation rate.

Here, we illustrate that masturbation is not simply a pathological behaviour, and is unlikely to solely be a byproduct of high sexual arousal. We provide the first evidence that both postcopulatory selection pressure and pathogen avoidance may influence this common, but little understood, sexual behaviour at a macroevolutionary scale. Considering the behavioural and socioecological complexity of primate societies, it is likely that primates employ masturbation as a flexible strategy according to the circumstances they find themselves in.

## 4. Methods

While the taxonomic classification of primates is subject to constant revision (e.g. [38]), the taxa in our analyses reflect the extant species present in the 10kTrees GenBank phylogenies [23]. In rare circumstances when data were given to subspecies level, these were grouped as a single species.

### (a) Masturbation data

We define masturbation as the self-stimulation of the anogenital or breast region, carried out with an individual's own body parts or external tools [1,2]. This operational definition treats internal motivations as a black box, albeit without ascribing to the stance that mental causes are absent.

Data were compiled from the literature, as well as questionnaire responses. Particular care was taken to solicit information from primatologists and zoo-keepers who had extensive experience with taxa for which published literature provided little or no information about masturbation (details in [1,2]). Species with a single confirming scientific report were considered to be masturbatory. Thus, reports of masturbation presence overrode those of masturbation absence, regardless of the number of times absence was reported (i.e. if 10 people said a species did not masturbate, but one individual confirmed they did, this species would be classed as masturbatory). Conversely, a species was not simply classified as non-masturbatory if there were a lack of reports on the behaviour, because behavioural studies of many primate species are limited, and masturbation may be rare, so anecdotal incidences are unlikely to be published. Instead, we only included cases which explicitly state that the behaviour was not observed.

### (b) Postcopulatory selection data

Primate species were classified according to whether their mating system and anatomy were likely to be associated with high or low postcopulatory competition. In the first case, species with single-male mating systems (monogamy, polygyny) were considered as having low postcopulatory competition, while species with multi-male mating systems (polygynandry, polyandry) were considered to have high postcopulatory competition, following Brindle & Opie [24]. Species with intraspecific variability that routinely spans both sides of the binary grouping were discarded from the analyses. In the second case, species with larger adult male testes mass (while controlling for body size) were identified as experiencing higher postcopulatory selection [25]. Mating system (single-male versus multi-male; *n*_species_ = 181) and testes mass (*n*_species_ = 75), were therefore employed as proxies for postcopulatory selection pressure. These data were taken from Brindle & Opie [24].

### (c) Pathogen load data and environmental proxies

Two different methods were adopted to evaluate the risk of infection from sexually transmitted pathogens: a direct approach, in which we assessed the frequency with which species were recorded to carry sexually transmitted pathogens; and an indirect approach, in which we assessed environmental correlates of pathogen abundance. Data on environmental measures were available for a wide sample of primate species [39], while the sample for sexually transmitted pathogens was less extensive [28].

In the first approach, a measure of sexually transmitted pathogen load in wild populations was gleaned from the Global Mammal Parasite Database v. 2.0 (GMPD; note that here ‘parasite' also refers to viral, bacterial, and fungal pathogens [40]). Data were filtered to include only sexually transmitted pathogens, and only those primate species included in the 10k Trees GenBank phylogenies. Data in which primates were only labelled to genus were also removed. While it is unlikely that pathogens are truly absent across a species, for the purposes of our analyses, we marked them as such if all reported pathogen screens were negative in at least 10 individuals (see electronic supplementary material for further discussion).

In the second approach, given the limited availability of data on pathogen load, we assessed the environmental correlates of pathogen infection risk. In the light of previous studies (e.g. [26,27]), we identified three potential predictors. These comprised (i) geographical region (temperate versus tropical; *n*_species_ covered = 7 and 117, respectively), (ii) mean annual precipitation (*n*_species_ covered = 147) and (iii) environmental harshness (*n*_species_ covered = 147) as proxies for pathogen load. These data were collected from Botero *et al*. [39], who measure precipitation as the mean annual precipitation averaged across a species' geographical range, and environmental harshness as the amount of exposure to drier, less productive environments, with colder, and more variable annual temperatures (with higher scores indicating harsher environments). Only data for primate species included in the 10k Trees GenBank phylogenies were collated from the database. Analyses of the environmental correlates were restricted to only include species for which data were available for all variables within a model.

### (d) Phylogenies

Comparative analyses must incorporate phylogenetic information to control for the confounding effects of shared ancestry between species, given that cross-taxa data are not statistically independent [41]. We therefore conducted phylogenetic analyses across a posterior distribution of 10 000 molecular phylogenies following GenBank taxonomy, from the 10k Trees Project v. 3.0 [23]. Where a single tree was required, we generated a maximum clade credibility tree from our distribution of 10 000 molecular ultrametric phylogenies, using TreeAnnotator (part of the BEAST software package [42]).

### (e) Phylogenetic signal

The phylogenetic signal of female and male masturbation was calculated using the ‘phylo.d' function within the R package ‘caper' [43,44].

### (f) Ancestral state reconstructions

We used a Bayesian MCMC framework to reconstruct the evolutionary history of masturbation across the primate order for females and males, using the ‘MultiState' function in BayesTraits v. 3.0 [45]. The analyses were carried out at the species level, with missing data included as such, so that nodes could take one of two states (masturbation present versus masturbation absent). A reversible-jump hyperprior approach was employed, seeding from an exponential distribution with a range of 0.00–0.05. Hyperpriors allow the details of the prior distribution to be estimated from the data, rather than an *a priori* assumption, which is useful where investigators have little or no information about the mean and variance of rate coefficients [46]. Each model was run three times, and the results were checked for equivalence. We then selected the model with the median log marginal likelihood (following [47]). Models were run for 5 000 000 iterations, with a burn in of 50 000 iterations. Ancestral states of interest were reconstructed via the ‘Add MRCA’ function in BayesTraits v. 3.0 [45]. The nodes reconstructed were the most recent common ancestors of (A) all primates, (B) strepsirrhines, (C) haplorrhines, (D) haplorrhines excluding tarsiers, (E) platyrrhine monkeys, (F) catarrhines, (G) apes and (H) catarrhine monkeys. Using this approach, the probability of a given state at any one node is generated. We consider probabilities > 0.70 as providing high confidence of a given state, > 0.60 as low confidence and < 0.60 as equivocal (following [47]).

### (g) Correlated evolution

Two binary traits (such as masturbation presence versus absence, and single-male versus multi-male mating systems) can produce four different combinations of observable states within a species across a phylogeny. If traits have coevolved, the rate of change between two states should depend on the background state of the other [46]. If the traits followed an independent pattern of evolution, the rate of change of one state should be independent of the other. By combining information on ancestral states with posterior rate distributions (i.e. transition rates), it is possible to discern the probable coevolutionary pathway two traits have taken [46]. We tested for coevolution between (i) masturbation and mating system and (ii) masturbation and pathogen presence, in both female and male primates. In addition, we checked for coevolution between mating system and pathogen presence, to exclude the possibility that the independent variables were influencing one another.

To do this, we employed reversible-jump Markov chain Monte Carlo (rjMCMC) techniques in BayesTraits v. 3.0 [46] to simultaneously test for coevolution between binary variables and estimate transition rates between states. This method fits continuous-time Markov models to discrete traits, allowing them to transition to another state at any time. The rjMCMC visits each model in proportion to its posterior probability, searching for the best-fitting model to describe the coevolution of two traits across a phylogeny. We compared the fit of a dependent model of evolution, in which transition rates were free to vary, switching ‘on' and ‘off', to that of an independent model, where transition rates between states were constrained to be equal. Transition rates were estimated by taking the mean posterior rate estimate across all posterior models for which a given transition was active. We also calculated the proportion of iterations in which each parameter was estimated to be zero (i.e. when a transition was ‘switched off') within the rjMCMC model.

To ensure these estimates were accurate and stable, with little run-to-run variation, each chain was run three times. If the chains converged well, the model with the median log marginal likelihood was chosen (following [47]). Markov chains were run for 5 000 000 iterations, after a burn-in of 500 000 samples. We employed a reversible-jump hyperprior approach, seeding from an exponential distribution of 0–0.5. Model fit was established using Log Bayes Factors (BFs) = 2(m_D_–m_I_). Natural log marginal likelihoods of the dependent (m_D_) and independent (m_I_) models were estimated using a stepping-stone sampler set with 100 stones, each run for 10 000 iterations, and the default parameters of *α* = 0.4 and *β* = 1. BFs were interpreted following Kass & Raftery [48]: 0–2, minimal support; 2–6, positive support; 6–10, strong support; greater than 10, very strong support.

### (h) Phylogenetic logistic regressions

We built Bayesian phylogenetic binary logistic regression models using the *brms* package in R [44,49]. We modelled masturbation presence as a binary response (present versus absent) separately for two different environmental fixed effects (mean annual precipitation and environmental harshness), and one postcopulatory fixed effect (testes mass). All analyses were run for female and male primates separately, resulting in four environmental models and two postcopulatory models. We accounted for phylogenetic relationships in all models by including a variance-covariance matrix of phylogenetic distance as a random effect. We included adult male body mass as a fixed effect in our postcopulatory models to control for the effects of body size on testes mass, rather than using residuals from a separate regression between the two variables as data, which can lead to biased parameter estimates [50].

We ran four chains for 10 000 iterations, with a burn-in of 5000 iterations using default priors. Delta was adapted to 0.9999 and maximum tree depth set to 12. Model performance was evaluated by visually assessing trace plots as well as examining R-hat values (with those <1.01 considered to have mixed well) and effective sample sizes (Bulk ESS should be greater than 100 times the number of chains, so in our case greater than 400). We considered statistical differences to be substantial if the 95% Bayesian credible intervals did not overlap zero, and moderate if 80% Bayesian credible intervals did not overlap zero.

## Data Availability

The data and code are provided in the electronic supplementary material [51].
